# Nationwide Study of the Outcome of Treatment of Lower Extremity Atherosclerotic Lesions With Endovascular Surgery With or Without Drug Eluting Methods in Patients With Diabetes

**DOI:** 10.1177/15266028241241967

**Published:** 2024-04-05

**Authors:** Torbjörn Fransson, Andrea Dahl Sturedahl, Timothy Resch, Eliasson Björn, Anders Gottsäter

**Affiliations:** 1 Department of Clinical Sciences, Lund University, Malmö, Sweden; 2 Vascular Center, Department of Thoracic and Vascular Surgery, Skåne University Hospital, Malmö, Sweden; 3 National Diabetes Register, Department of Data Management and Analysis, Region Västra Götaland, Gothenburg, Sweden; 4 Department of Vascular Surgery, Copenhagen University Hospital, Copenhagen, Denmark; 5 Faculty of Health Sciences, Copenhagen University, Copenhagen, Denmark; 6 Department of Medicine, Sahlgrenska University Hospital, Gothenburg, Sweden; 7 Department of Medicine, Skåne University Hospital, Malmö, Sweden

**Keywords:** intermittent claudication, diabetes mellitus, endovascular treatment/therapy, drug-coated balloon, drug eluting balloon, drug eluting stent, chronic limb threatening ischemia, drug-coated stent

## Abstract

Paclitaxel eluting balloons and stents reduce the development of intimal hyperplasia and restenosis rate after endovascular treatment for peripheral arterial disease (PAD) in the lower extremities. As diabetes mellitus (DM) worsens the prognosis in PAD patients after invasive endovascular treatment, we assessed the effects of drug eluting technology for PAD with special focus on effects in patients with and without DM. All Swedish patients registered in the Swedish vascular registry Swedvasc after vascular or endovascular surgery for intermittent claudication, IC; (n=1948) or chronic limb threatening ischemia, CLTI (n=3353) in 2013 to 2015 were followed up regarding a composite of either major amputation and/or mortality during median 607 days and for reinterventions for PAD during median 522 days. Patients with DM registered in the Swedish National Diabetes Registry (NDR) and patients without DM were evaluated separately. The 201 CLTI patients with DM treated with drug eluting methods had a lower adjusted risk for amputation or mortality after adjustment than the 1476 CLTI patients with DM treated without drug eluting methods (hazard ratio [HR] 0.712 [0.562-0.901], *P*=.005). There were no differences among IC patients. Regarding the need for reinterventions for PAD, there were no differences between patients treated with and without drug eluting methods, irrespectively of the presence or absence of DM. In conclusion, this nationwide follow-up study indicated potential benefits of drug eluting treatment in CLTI patients with diabetes. The results need to be evaluated and confirmed in prospective randomized studies.

**Clinical Impact:**

This retrospective observational registry trial combines national registries for vascular surgical procedures and diabetes mellitus to clarify results of drug eluting technology in treating diabetic subjects with intermittent claudication or chronic limb threatening ischemia compared to treatment of non-diabetic subjects. As earlier proposed and showed in this trial, there may be an implication for a beneficial treatment efficacy with drug eluting therapy in the diabetic population with PAD compared to the non-diabetic population. A finding worth further exploration.

## Introduction

Diabetes mellitus (DM) affects more than 450 million people worldwide and is an increasing burden,^1,2^ causing atherosclerosis through effects on platelets, endothelial cell and smooth muscle cell function.^3^ These effects make DM one of the leading risk factors for atherosclerotic disease in peripheral arteries in the lower extremities (PAD).^1,3–5^ This disease can manifest itself as intermittent claudication (IC) or critical limb threatening ischemia (CLTI) with rest pain or ulceration.^6,7^ Both conditions are associated with increased long-term mortality.^8,9^ Diabetes mellitus also has a negative impact on the long-term prognosis regarding major cardiovascular events (MACEs), acute myocardial infarction (AMI), and major amputation after invasive endovascular treatment of both IC and CLTI.^10–24^

Drug eluting treatment (DET) with balloon angioplasty (drug eluting balloons, DEBs) and/or stents (drug eluting stents, DESs) coated with the antiproliferative drug paclitaxel have beneficial effects on the rate of restenosis by reducing the development of intimal hyperplasia and are considered safe.^15,25–36^

All Swedish patients undergoing surgery for IC and CLTI are registered in the Swedish vascular registry (Swedvasc).^37^ Preoperative symptoms and risk factors are registered together with the type of treatment (acute or elective, anatomic disease location, open, endovascular, or hybrid treatment), and patients are routinely followed up after 1- and 12-months regarding survival, amputation, or complications.

All DM patients in Sweden are registered in the Swedish National Diabetes Registry (NDR),^38^ reporting on treatment and risk factor control based on information from medical records at least once a year at hospital outpatient and primary health care clinics.

The study aim was to evaluate whether PAD patients, with and without DM, who have undergone endovascular treatment for PAD with drug eluting technologies have a better outcome than patients treated without the use of drug eluting technology. The primary endpoint was a composite of either major amputation and/or mortality or reinterventions for PAD.

## Materials and Methods

In this nationwide observational cohort study, data from Swedvasc were linked to baseline and follow-up data from national registries and the NDR. Outcome variables are registered in Swedvasc in accordance with the International Consortium of Vascular Registries consensus recommendations for peripheral revascularisation registry data collection,^39^ and the study reports objective performance goals recommended by the Society for Vascular Surgery for reporting on CLTI.^40^

**Figure 1.**
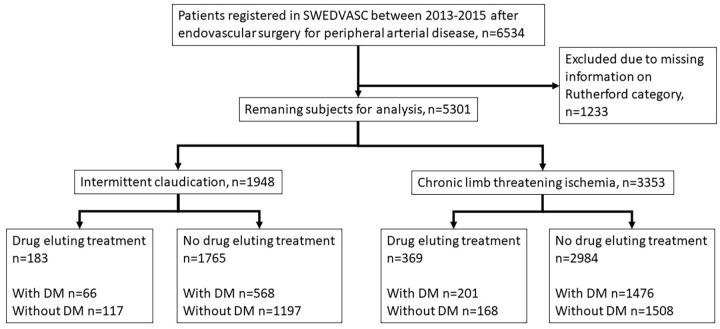
Consort flow chart of patients in the Swedish Vascular Register (Swedvasc) undergoing endovascular vascular surgery for intermittent claudication or chronic limb threatening ischemia with or without the use of drug eluting technologies. Separate analysis of patients with and without diabetes in the different groups. DM=diabetes mellitus.

All patients registered in the Swedvasc PAD module 2013 to 2015 after endovascular treatment for IC or CLTI were included (Figure 1). Patients in whom information on Rutherford category (RC) was missing (n=1233) were excluded. The remaining population was subdivided regarding indication for treatment; IC (n=1948) or CLTI (n=3353). Subpopulations treated with drug eluting methods in the IC and CLTI groups were defined, whereafter patients treated with and without drug eluting methods were compared separately both to those with a concomitant registration in the NDR (i.e., patients with DM) and those without such registration (i.e., patients without DM; Figure 1). In conclusion, a total of 5301 subjects were analyzed and stratified for IC and CLTI, presence of DM or not, and treatment with drug eluting methods or not. For multilevel treatment with mixed drug eluting and no drug eluting therapy, subjects were analyzed in the drug eluting therapy (DET) group.

Collection of registry data has been described in detail before.^10–12,41^ Baseline data were retrieved from the inpatient registry (IPR) administered by the National Board of Health and Welfare (http://socialstyrelsen.se/english)^42^ with nationwide data for primary and secondary discharge diagnoses and lengths of hospitalization since 1987,^42^ the Prescribed Drug Register (PDR) with full information about filled prescriptions since 2005,^43^ and the cancer registry (http://socialstyrelsen.se/english). All these registries were used for information about comorbidities and drug treatment at baseline.

The IPR uses International Classification of Diseases (ICD): Revision 10, for classification of diagnoses. Comorbidities at baseline included AMI, congestive heart failure (CHF), atrial fibrillation or flutter (AF), hypertension, coronary heart disease (CHD), stroke, and any cardiovascular disease defined as CVD as AMI and/or stroke prior to index date. At baseline, we also included psychiatric disorders, cancer, liver disease, chronic obstructive pulmonary disease (COPD), and renal disorders. All ICD codes registered at baseline are listed in Supplementary Table S1.

Data on use of aspirin, antihypertensive, lipid lowering, and anticoagulant therapy at baseline was retrieved from the PDR. Hypertension was defined as one prescription for antihypertensive medication during the year prior to index operation date. One prescription is equivalent to 3 months of continuous use of a drug. The most common antihypertensive medication in Sweden at the time of the study were: angiotensin-converting-enzyme (ACE) inhibitors, diuretics, calcium channel blockers (CCB), angiotensin II-receptor blockers (ARB), alfa-1 receptor blockers, beta blockers, and various combinations of the above.

Mortality was followed up until December 31, 2017, using the Cause of Death Register^44^ with complete information on the causes and time of death (http://socialstyrelsen.se/english). The IPR^42^ was used for follow-up of CV morbidity, major adverse cardiovascular events (MACEs), defined as ischemic heart disease (ICD codes I20-I25), cerebrovascular disease (ICD codes I61-I64), coronary revascularization (procedure codes FN), and major amputations until December 31, 2016.

Median time of follow-up (defined as median number of days until censoring or until an event occurred) was 607 days for the composite of either major amputation or mortality, and 522 days for PAD reinterventions. Median values regarding duration of follow-up for secondary endpoints are presented in Supplementary Table S2.

Information regarding stratification of anatomical location or modality of treatment (stent or balloon only) is shown in Supplementary Tables S3 and S4.

### Primary Outcomes

Comparison of PAD patients treated endovascularly with and without the use of drug eluting technologies regarding a composite of either major amputation and/or mortality, and regarding PAD reintervention. The outcomes were separately analyzed in patients with and without DM.

### Secondary Outcomes

Comparison of patients treated with endovascular surgery for IC or CLTI with and without the use of drug eluting technologies regarding all-cause mortality, cardiovascular mortality, major amputation, MACEs, myocardial infarction, and stroke. The outcomes were separately analyzed in patients with and without DM.

### Statistical Methods

Descriptive statistics are presented as mean and standard deviation for numerical variables and count and percentages for categorical variables, unless stated otherwise. Primary and secondary outcomes were examined using incidence rates with 95% Poisson confidence intervals. We constructed crude Kaplan-Meier curves and performed Cox regression adjusted for the following variables at baseline: age, sex, smoking, any cardiovascular disease, lipid lowering treatment, aspirin, and oral anticoagulants. Multiple imputations with logistic regression, 20 imputations and 15 iterations were used for missing values in the smoking variable.

Owing to the low number of secondary outcome events, separate Kaplan-Meier curves and Cox regression analyses of total mortality and amputation were only calculated for CLTI patients. Major adverse cardiovascular event was only calculated for IC patients due to statistical problems with fulfilling the proportional hazards assumption. Regarding cardiovascular death, AMI, and stroke, no analyses were performed.

## Ethical Approval

The study was approved by the regional ethics research committee, (Dnr 2016/232 and Dnr 2016/544). Individual consent is not required to report patients to national quality registries of health care, or to be included in a study like this, according to Swedish law (Patient Data Act 2008:355, chapter 7).

## Results

Baseline characteristics for the variables included in the Cox models are presented in Table 1 for subjects with IC and in Table 2 for subjects with CLTI. Full background data for all patients are shown in Supplementary Tables S5 and S6. As expected, primary outcome events were few among IC patients. The number of patients with outcome events and incidence rates are presented in Tables 3 for patients with IC, and in Tables 4 for patients with CLTI.

**Table 1.** Baseline Characteristics of Variables Included in the Cox Models in Patients With Intermittent Claudication (IC) Undergoing Endovascular Treatment With or Without Drug Eluting Technologies, Subdivided in Groups With and Without Diabetes Mellitus.

Variables included in Cox models	Patients with diabetes mellitus		Patients without diabetes mellitus	
	DE methods	No DE methods	DE methods	No DE methods
N	66	568	117	1197
Smoking (%)	10 (15.4)	89 (15.7)	15 (14.4)	186 (16.9)
Age (mean [SD])	69.89 (8.55)	70.79 (8.13)	73.91 (8.55)	71.79 (8.72)
Male sex (%)	40 (60.6)	349 (61.4)	63 (53.8)	610 (51.0)
Cardiovascular disease (mean [SD])	0.47 (0.50)	0.54 (0.50)	0.39 (0.49)	0.36 (0.48)
Lipid lowering (mean [SD])	0.85 (0.36)	0.89 (0.31)	0.84 (0.37)	0.82 (0.38)
Aspirin (mean [SD])	0.74 (0.44)	0.78 (0.41)	0.81 (0.39)	0.79 (0.40)
Oral anticoagulant (mean [SD])	0.39 (0.49)	0.34 (0.47)	0.41 (0.49)	0.27 (0.45)

DE=drug eluting.

**Table 2.** Baseline Characteristics of Variables Included in the Cox Models in Patients With Chronic Limb Threatening Ischemia (CLTI) Undergoing Endovascular Treatment With or Without Drug Eluting Technologies, Subdivided in Groups With and Without Diabetes Mellitus.

Variables included in Cox models	Patients with diabetes mellitus		Patients without diabetes mellitus	
	DE methods	No DE methods	DE methods	No DE methods
N	201	1476	168	1508
Smoking (%)	31 (15.8)	249 (17.3)	24 (18.8)	331 (28.7)
Age (mean [SD])	73.88 (9.78)	75.22 (10.16)	79.55 (9.55)	79.26 (9.48)
Male sex (%)	126 (62.7)	892 (60.4)	76 (45.2)	605 (40.1)
Cardiovascular disease (mean [SD])	0.65 (0.48)	0.59 (0.49)	0.42 (0.49)	0.43 (0.50)
Lipid lowering (mean [SD])	0.79 (0.41)	0.69 (0.46)	0.60 (0.49)	0.56 (0.50)
Aspirin (mean [SD])	0.68 (0.47)	0.63 (0.48)	0.66 (0.47)	0.60 (0.49)
Oral anticoagulant (mean [SD])	0.47 (0.50)	0.45 (0.50)	0.42 (0.49)	0.40 (0.49)

DE=drug eluting.

**Table 3.** Number of Patients (Number Of Events) and Incidence Rates Per 1000 Person Years (95% Poisson Confidence Intervals) in Patients With Intermittent Claudication (IC) With and Without Diabetes Mellitus Treated With and Without Drug Eluting Methods.

Outcome	Patients with diabetes mellitus		Patients without diabetes mellitus	
	DE methods	No DE methods	DE methods	No DE methods
Amputation or mortality	66 (7) 57.8 (42.0-119.1)	568 (64) 59.2 (54.0-75.5)	117 (5) 24.0 (16.2-56.1)	1197 (93) 39.3 (36.5-48.1)
Reintervention for PAD	66 (9) 78.4 (59.6-148.9)	568 (100) 101.7 (94.7-123.8)	117 (18) 98.7 (82.2-156.1)	1197 (196) 92.9 (88.4-106.9)
Total mortality	66 (4) 31.7 (20.1-81.1)	568 (75) 68.0 (62.6-85.3)	117 (9) 42.8 (32.5-81.2)	1197 (110) 46.0 (43.0-55.5)
Cardiovascular death	66 (1) 7.8 (2.2-43.3)	568 (5) 4.3 (2.9-10.1)	117 (2) 9.5 (4.6-34.3)	1197 (14) 5.7 (4.6-9.6)
Amputation	66 (4) 31.7 (20.1-81.2)	568 (15) 13.1 (10.7-21.7)	117 (0) 0 (0-17.3)	1197 (13) 5.3 (4.3-9.1)
AMI	66 (4) 32.4 (20.5-82.9)	568 (30) 26.6 (23.2-37.9)	117 (0) 0 (0-17.3)	1197 (48) 20.0 (18.0-26.5)
MACE	66 (22) 222.2 (188.7-336.5)	568 (215) 264.6 (252.2-302.5)	117 (29) 164.3 (142.8-235.9)	1197 (323) 162.9 (156.7-181.8)
Stroke	66 (2) 15.6 (7.5-56.5)	568 (31) 27.5 (24.1-39.1)	117 (8) 39.6 (29.5-78.1)	1197 (35) 14.4 (12.7-20.1)

DE=drug eluting, AMI=acute myocardial infarction, MACE=major adverse cardiovascular events, PAD=peripheral arterial disease.

**Table 4.** Number of Patients (Number of Events) and Incidence Rates Per 1000 Person Years (95% Poisson Confidence Intervals) in Patients With Chronic Limb Threatening Ischemia (CLTI) With or Without Diabetes Mellitus Treated With and Without Drug Eluting Methods.

Outcome	Patients with diabetes mellitus		Patients without diabetes mellitus	
	DE methods	No DE methods	DE methods	No DE methods
Amputation or mortality	201 (77) 283.8 (261.4-354.7)	1476 (747) 405.3 (395.2-435.4)	168 (53) 220 (199-287.8)	1508 (607) 277.8 (270.1-300.8)
Reintervention for PAD	201 (44) 144 (128.9-193.4)	1476 (345) 141 (135.8-156.7)	168 (31) 122.8 (107.3-174.3)	1508 (366) 150.6 (145.2-166.8)
Total mortality	201 (63) 184.5 (168.3-236.0)	1476 (609) 253 (246.1-274.0)	168 (47) 175.4 (157.5-233.2)	1508 (553) 218 (211.7-236.9)
Cardiovascular death	201 (1) 2.6 (0.8-14.6)	1476 (34) 11.2 (9.9-15.7)	168 (1) 3.3 (0.9-18.2)	1508 (35) 11.5 (10.1-15.9)
Amputation	201 (43) 139.1 (124.2-187.3)	1476 (384) 164.5 (158.7-181.8)	168 (27) 98.9 (85.5-144.0)	1508 (245) 92.8 (88.7-105.2)
AMI	201 (16) 43.7 (35.9-71.0)	1476 (151) 52.8 (49.8-61.9)	168 (8) 26.8 (19.9-52.8)	1508 (86) 28.8 (26.6-35.6)
MACE	201 (98) 405.0 (376.7-493.5)	1476 (650) 331.1 (322.3-357.6)	168 (57) 251.9 (228.6-326.3)	1508 (465) 197.5 (191.3-216.3)
Stroke	201 (11) 29.6 (23.2-53.0)	1476 (99) 33.7 (31.4-41.0)	168 (9) 30.4 (23.1-57.7)	1508 (104) 35.2 (32.8-42.7)

DE=drug eluting, AMI=acute myocardial infarction, MACE=major adverse cardiovascular events, PAD=peripheral arterial disease.

**Figure 2.**
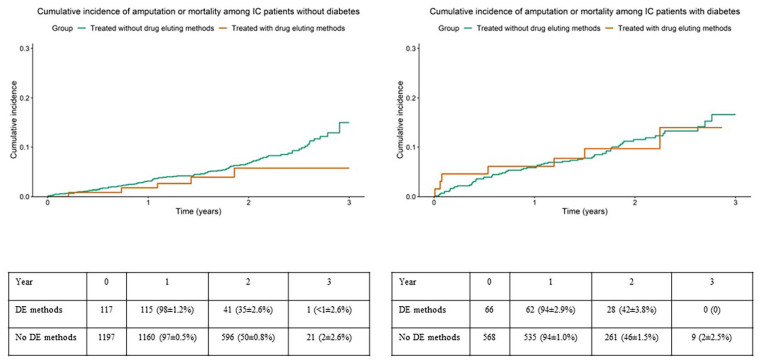
Crude rates of the composite variable amputation or mortality among patients with intermittent claudication, treated with and without drug eluting methods in subgroups without (left panel) or with (right panel) diabetes mellitus. Table showing limbs at risk at different points as numbers and %±SE. DE=drug eluting.

**Figure 3.**
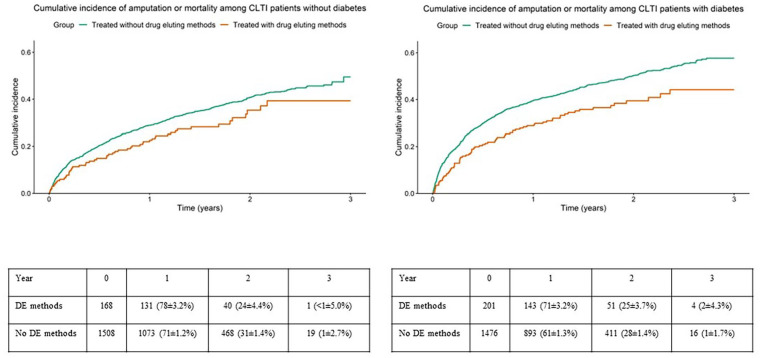
Crude rates of the composite variable amputation or mortality among patients with chronic limb threatening ischemia, treated with and without drug eluting methods in subgroups without (left panel) or with (right panel) diabetes mellitus. Table showing limbs at risk at different points as numbers and %±SE. DE=drug eluting.

**Figure 4.**
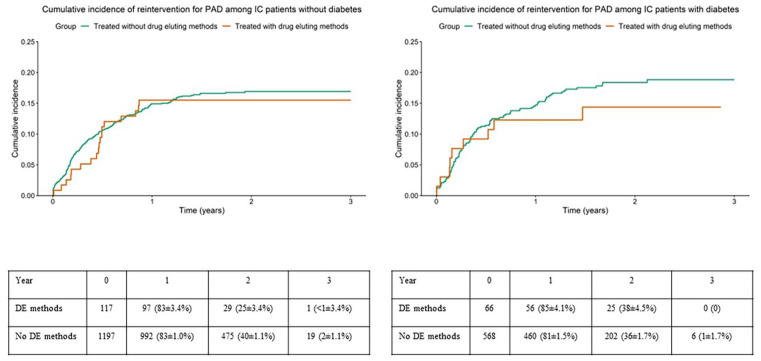
Crude rates of reinterventions for peripheral arterial disease among patients with intermittent claudication, treated with and without drug eluting methods in subgroups without (left panel) or with (right panel) diabetes mellitus. Table showing limbs at risk at different points as numbers and %±SE. DE=drug eluting.

**Figure 5.**
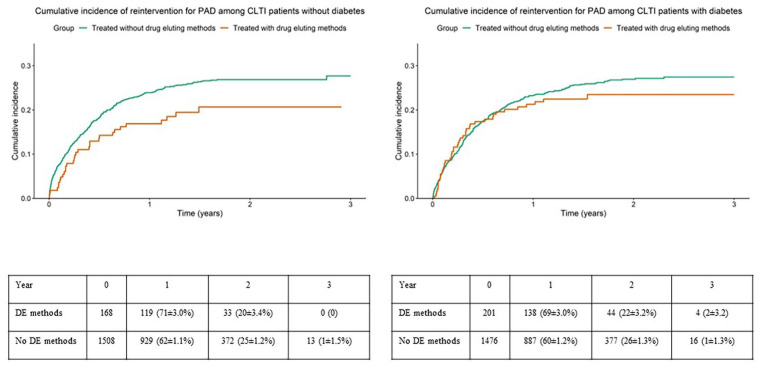
Crude rates of reinterventions for peripheral arterial disease among patients with chronic limb threatening ischemia, treated with and without drug eluting methods in the subgroups without (left panel) or with (right panel) diabetes mellitus. Table showing limbs at risk at different points as numbers and %±SE. DE=drug eluting.

Crude rates of the composite variable amputation or mortality treated with and without drug eluting methods in subgroups without or with diabetes are shown in Figure 2 for IC patients and in Figure 3 for CLTI patients. Crude rates of reinterventions for PAD treated with and without drug eluting methods in the subgroups without or with diabetes are shown in Figure 4 for IC patients and in Figure 5 for CLTI patients.

Results of unadjusted and adjusted analyses regarding amputation or mortality and reintervention for PAD during follow-up among IC patients with and without DM are presented in Table 5 and the corresponding results for CLTI patients in Table 6. Whereas, there were no differences among IC patients, CLTI patients with DM treated with drug eluting methods had a lower risk for amputation or mortality after adjustment compared to patients treated without drug eluting methods (hazard ratio [HR]: 0.712 [0.562-0.901], *P*=.005, Table 6, Figure 3). There were no differences between IC or CLTI patients treated with and without drug eluting methods regarding the need for reintervention for PAD, irrespectively of the presence or absence of DM (Tables 5 and 6, Figures 4 and 5).

**Table 5.** Hazard Ratio (HR) With 95% Confidence Intervals (CI) for Patients Treated With Drug Eluting Methods Compared to Those Treated Without Drug Eluting Methods Among Patients With Intermittent Claudication (IC) With and Without Diabetes, Respectively.

Model	HR (95% CI)	*P* value	Outcome	Subgroup
Adjusted	1.074 (0.482-2.390)	.859	Amputation or mortality	IC with diabetes
Unadjusted	0.979 (0.442-2.168)	.958	Amputation or mortality	IC with diabetes
Adjusted	0.540 (0.215-1.354)	.186	Amputation or mortality	IC without diabetes
Unadjusted	0.646 (0.259-1.609)	.345	Amputation or mortality	IC without diabetes
Adjusted	0.760 (0.380-1.521)	.434	Reintervention for PAD	IC with diabetes
Unadjusted	0.766 (0.384-1.527)	.445	Reintervention for PAD	IC with diabetes
Adjusted	0.877 (0.538-1.430)	.598	Reintervention for PAD	IC without diabetes
Unadjusted	0.937 (0.577-1.522)	.791	Reintervention for PAD	IC without diabetes

PAD=peripheral arterial disease.

**Table 6.** Hazard Ratio (HR) With 95% Confidence Intervals (CI) For Patients Treated With Drug Eluting Methods Compared to Patients Treated Without Drug Eluting Methods Among Patients With Chronic Limb Threatening Ischemia (CLTI) With and Without Diabetes, Respectively.

Model	HR (95% CI)	*P* value	Outcome	Subgroup
Adjusted	0.712 (0.562-0.901)	.005	Amputation or mortality	CLTI with diabetes
Unadjusted	0.698 (0.552-0.883)	.003	Amputation or mortality	CLTI with diabetes
Adjusted	0.797 (0.600-1.057)	.115	Amputation or mortality	CLTI without diabetes
Unadjusted	0.783 (0.591-1.037)	.088	Amputation or mortality	CLTI without diabetes
Adjusted	0.849 (0.619-1.165)	.309	Reintervention for PAD	CLTI with diabetes
Unadjusted	0.895 (0.654-1.227)	.490	Reintervention for PAD	CLTI with diabetes
Adjusted	0.702 (0.486-1.016)	.061	Reintervention for PAD	CLTI without diabetes
Unadjusted	0.715 (0.495-1.032)	.073	Reintervention for PAD	CLTI without diabetes

PAD=peripheral arterial disease.

Regarding the secondary outcomes as total mortality, amputation, and MACE (supplementary table S7), the total mortality was lower in patients with DM that received treatment with drug eluting technology, compared to those that were treated with standard endovascular technology. The difference was largest around 12 months (*P*=.001) of follow-up, and later followed by a “catch up” phenomenon. No other differences concerning secondary variables were seen between those treated with and without drug eluting methods among IC or CLTI patients with or without DM.

## Discussion

We found that CLTI patients with DM who were treated with drug eluting methods had a lower risk for amputation or death than patients treated without drug eluting methods, whereas we were not able to demonstrate any benefit among CLTI patients without DM or among IC patients.

The most effective type of endovascular treatment for lower extremity PAD still remains to be determined,^25,34,36,45–47^ but the use of drug eluting technology might be an attractive adjunct to improve outcomes, in particular, as DM has an adverse effect on the prognosis after endovascular intervention,^10–12,15,16,18,21,24,48–50^ drug eluting technologies might potentially offer extra benefits in this subpopulation.

Diabetic patients have constituted 60% to 100% of the material in previous studies of drug eluting therapy in CLTI,^25^ and their rate of cardiovascular events after revascularization is higher than in CTLI patients without DM.^51^ Furthermore, their PAD lesions are more often more complex.^48^ Even if the role of DM as a predictor of restenosis is not clear,^52^ the presence of diabetic foot ulcers negatively affects both amputation rates as well as overall survival in this patient cohort, and thus, improved therapies are of paramount importance. The presence of vascular inflammation as well as increased smooth muscle migration and proliferation can perhaps be targeted by paclitaxel, and this is a possible mechanism to account for the improved outcomes in diabetic CLTI patients.^53^

One important study limitation is the fact that we performed a nonrandomized comparative cohort study to elucidate a research question which would have needed a randomized study to be conclusively answered. Such a study, the Swedepad^29^ is ongoing and will offer new important information within the next years.

The DET group was smaller than the group receiving standard treatment, and confounding might have been caused by differences in treatment region and treatment complexity despite that the calculations were adjusted for other demographic factors; however, in an observational, nonrandomized study selection bias might always result in imbalance between groups regarding factors affecting the outcome, whether group sizes are balanced or not.

Adjustment of the data for anatomical location or modality was not feasible due to limited numbers. We primarily wanted to evaluate diabetic and nondiabetic as well as IC and CLTI patients separately. Too many additional adjustment variables would have introduced a high risk of an overfitted statistical model with a higher risk of multicollinearity, resulting in low generalizability and risk of inaccurate estimates, confidence intervals, and *P* values. Therefore, only the adjustment variables that were considered the most important were selected as covariates.

Furthermore, as patients had been treated at different Swedish vascular centers with varying institution and operator case load, several different types of balloons and stents had been used. This constitutes a theoretical reason for hampered technical results and treatment efficacy. The fact that the number of patients in some of the different subgroups were small also limited our possibilities to perform reliable statistical analyses, especially regarding the secondary outcomes. This might also explain the fact that we were not able to demonstrate any benefit among CLTI patients without DM or among IC patients.

Another limitation may be that we in some ways must bear in mind that the endovascular technology regarding drug-coated balloons and DESs has evolved significantly since the years 2013 to 2015 as also the operators probably today have even further knowledge regarding feasibility and operational skills.

It is clearly of paramount importance to separately analyze patients with IC and CLTI due to the profound differences regarding general outcome effects and relevant outcome variables, between these 2 groups.

The fact that the study was nationwide constitutes important study strength. Swedish national registries are reliable regarding reporting of hospitalization, death, and reinterventions. Although Swedvasc collects and reports data in accordance with current reporting guidelines,^39,40^ some performance data are not collected. For example, detailed information regarding ulcers, healing, and functional performance status is not available, limiting interpretation of detailed outcome efficacy especially among subjects with diabetes. Our choice of endpoint variables seems relevant, also when compared with recently published trial protocols for CLTI.^29,54^ The accuracy of Swedvasc has been systematically evaluated regarding procedures for carotid artery disease and abdominal aortic aneurysm,^55^ but though not regarding endovascular treatment of IC and CLTI. Misclassification of these 2 entities in the registry has been reported^56^ and might have occurred also in our study. We also adjusted for several important confounders, such as age, sex, smoking, previous cardiovascular disease, lipid lowering treatment, aspirin, and oral anticoagulants. On the contrary, potential actual group differences in lipid and blood pressure levels were not accounted for.

Subjects undergoing solitary treatment in the aorto-iliac region usually suffer from IC and not CLTI, and patency rates at this level outperform patency rates of treatment at lower levels.^57,58^ We have chosen to *not* exclude this group as the number of subjects receiving DET at this level were extremely few, only 2 with CLTI and 7 with IC. These numbers will not affect the outcomes in favor of DET, instead they might by potential dilutional statistical effects make the performance in the non-DET group look somewhat better. As the primary outcomes in our study for all regions were in favor of DET, exclusion of this group did not seem relevant.

Duration of follow-up is comparably long in this study. Most similar studies have follow-up periods of 12 months or less, although there are published randomized studies regarding CLTI patients with longer follow-up.^28,35,59–65^

Furthermore, it is important to note that potential benefits of drug eluting technology are only a possible adjunct to the multidisciplinary approach including blood glucose and risk factor control, appropriate wound care, offloading of foot ulcers and necessary control of leg edema to improve limb salvage and mortality in PAD patients with diabetes.^66^

In conclusion, this nationwide follow-up study suggests potential benefits of DET in CLTI patients suffering from DM. The results need to be evaluated and confirmed in prospective randomized studies.

## Supplemental Material

sj-docx-1-jet-10.1177_15266028241241967 – Supplemental material for Nationwide Study of the Outcome of Treatment of Lower Extremity Atherosclerotic Lesions With Endovascular Surgery With or Without Drug Eluting Methods in Patients With Diabetes Supplemental material, sj-docx-1-jet-10.1177_15266028241241967 for Nationwide Study of the Outcome of Treatment of Lower Extremity Atherosclerotic Lesions With Endovascular Surgery With or Without Drug Eluting Methods in Patients With Diabetes by Torbjörn Fransson, Andrea Dahl Sturedahl, Timothy Resch, Eliasson Björn and Anders Gottsäter in Journal of Endovascular Therapy
