# Supplementary material for: Nationwide Study of the Outcome of Treatment of Lower Extremity Atherosclerotic Lesions With Endovascular Surgery With or Without Drug Eluting Methods in Patients With Diabetes
Source: J Endovasc Ther. 2024 Apr 5;32(6):2196–207. doi: 10.1177/15266028241241967 (PMC12598076; doi:10.1177/15266028241241967)
Supplement: sj-docx-1-jet-10.1177_15266028241241967 – Supplemental material for Nationwide Study of the Outcome of Treatment of Lower Extremity Atherosclerotic Lesions With Endovascular Surgery With or Without Drug Eluting Methods in Patients With Diabetes [file sj-docx-1-jet-10.1177_15266028241241967.docx]

| Supplementary table S1. Concomitant diseases at baseline, ICD-10 codes. | | |
| --- | --- | --- |
| Acute myocardial infarction | I21…, |  |
| Coronary heart disease | I20…, I21…, I22…, I23…, I24…, I25…, |  |
| Stroke | I61…, I62…, I63…, I64…, |  |
| Cerebrovascular disease | I21…, I61…, I62…, I63…, I64…, |  |
| Atrial fibrillation | I48…, |  |
| Congestive heart failure | I50…, |  |
| Renal disorders | N17…, N18…, N19…, N99…, V42A, V45B, V56A, V56W, Z940, Z491, Z492, Z992 |  |
| Cancer | C0…, C1…, C2…, C3…, C4…, C5…, C6…, C7…, C8…, C9…, |  |
| Liver disorders | K70…, K71…, K72…, K73…, K74…, |  |
| Psychiatric disorders | F20…, F21…, F22…, F23…, F24…, F25…, F26…, F27…, F28…, F29…, F30…, F31…, F32…, F33…, F34…, F35…, F36…, F37…, F38…, F39… |  |
| Chronic obstructive pulmonary disease | J44…, |  |

Supplementary table S2. Median follow up in days with interquartile range, for primary and secondary outcomes. AMI = acute myocardial infarction, MACE = major adverse cardiovascular events, PAD = peripheral arterial disease.

| Outcome | Median follow up (days) |  |
| --- | --- | --- |
| Amputation or mortality | 607 (390-835) |  |
| Reintervention for PAD | 522 (231-780) |  |
| Total mortality | 662 (453-870) |  |
| Cardiovascular death | 746 (565-933) |  |
| Amputation | 601 (383-831) |  |
| MACE | 662 (453-870) |  |
| AMI | 639 (432-851) |  |
| Stroke | | 640 (434-851) |

Supplementary table S3. Patients with intermittent claudication stratified for anatomical level and modality, presented as numbers and percentages.

|  | Patients with diabetes mellitus | | Patients without diabetes mellitus | |
| --- | --- | --- | --- | --- |
|  | **DE methods** | **No DE methods** | **DE methods** | **No DE methods** |
| N | 66 | 568 | 117 | 1197 |
| Aortoiliacal n (%) | 2 (3.0) | 236 (41.5) | 5 (4.3) | 567 (47.4) |
| Femoropopliteal n (%) | 53 (80.3) | 277 (48.8) | 88 (75.2) | 526 (43.9) |
| Infrapopliteal n (%) | 11 (16.7) | 55 (9.7) | 24 (20.5) | 104 (8.7) |
| Treated with stent n (%) | 27 (40.9) | 387 (68.1) | 40 (34.2) | 863 (72.1) |
| Treated without stent n (%) | 39 (59.1) | 181 (31.9) | 77 (65.8) | 334 (27.9) |

Supplementary table S4. Patients with chronic limb threatening ischemia stratified for anatomical level and modality, presented as numbers and percentages. DE = drug eluting.

|  | Patients with diabetes mellitus | | Patients without diabetes mellitus | |
| --- | --- | --- | --- | --- |
|  | **DE methods** | **No DE methods** | **DE methods** | **No DE methods** |
| N | 201 | 1476 | 168 | 1508 |
| Aortoiliacal n (%) | 0 (0.0) | 160 (10.8) | 2 (1.2) | 249 (16.5) |
| Femoropopliteal n (%) | 90 (44.8) | 576 (39.0) | 93 (55.4) | 727 (48.2) |
| Infrapopliteal n (%) | 111 (55.2) | 740 (50.1) | 73 (43.5) | 532 (35.3) |
| Treated with stent n (%) | 81 (40.3) | 632 (42.8) | 64 (38.1) | 810 (53.7) |
| Treated without stent n (%) | 120 (59.7) | 844 (57.2) | 104 (61.9) | 698 (46.3) |

Supplementary table S5. Baseline characteristics of patients with intermittent claudication (IC) undergoing endovascular treatment with or without drug eluting technologies, subdivided in groups with and without diabetes mellitus. DE=drug eluting, ACE = angiotensin converting enzyme, AMI = acute myocardial infarction, ARB = Angiotensin receptor blockers, COPD = chronic obstructive pulmonary disease, SD = standard deviation, USD = United States Dollars.

|  | Patients with diabetes mellitus | | Patients without diabetes mellitus | |
| --- | --- | --- | --- | --- |
|  | **DE methods** | **No DE methods** | **DE methods** | **No DE methods** |
| N | 66 | 568 | 117 | 1197 |
| Age (mean [SD]) | 69.89 (8.55) | 70.79 (8.13) | 73.91 (8.55) | 71.79 (8.72) |
| Male sex (%) | 40 (60.6) | 349 (61.4) | 63 (53.8) | 610 (51.0) |
| Smoking status (%) |  |  |  |  |
| Non-smoker | 55 (83.3) | 477 (84.0) | 89 (76.1) | 913 (76.3) |
| Smoker | 10 (15.2) | 89 (15.7) | 15 (12.8) | 186 (15.5) |
| Unknown | 1 (1.5) | 2 (0.4) | 13 (11.1) | 98 (8.2) |
| Civil status (%) |  |  |  |  |
| Married | 34 (51.5) | 289 (50.9) | 56 (47.9) | 544 (45.4) |
| Separated | 12 (18.2) | 131 (23.1) | 27 (23.1) | 322 (26.9) |
| Single | 11 (16.7) | 71 (12.5) | 10 (8.5) | 132 (11.0) |
| Widowed | 9 (13.6) | 77 (13.6) | 24 (20.5) | 199 (16.6) |
| Place of birth (%) |  |  |  |  |
| Sweden | 62 (93.9) | 459 (80.8) | 102 (87.2) | 1006 (84.0) |
| Europe except Sweden | 1 (1.5) | 41 (7.2) | 12 (10.3) | 106 (8.9) |
| Outside Europe | 3 (4.5) | 68 (12.0) | 3 (2.6) | 85 (7.1) |
| Income per month after tax (USD) (mean [SD]) | 1776.08 (1031.43) | 1878.22  (1513.49) | 2132.74 (1652.11) | 1946.09  (1210.13) |
| Concomitant diseases (mean [SD]) |  |  |  |  |
| AMI | 0.14 (0.35) | 0.21 (0.41) | 0.17 (0.38) | 0.14 (0.34) |
| Coronary heart disease | 0.39 (0.49) | 0.48 (0.50) | 0.35 (0.48) | 0.32 (0.47) |
| Stroke | 0.11 (0.31) | 0.12 (0.33) | 0.09 (0.29) | 0.09 (0.28) |
| Any cardiovascular disease | 0.24 (0.43) | 0.30 (0.46) | 0.23 (0.42) | 0.21 (0.40) |
| Atrial fibrillation | 0.17 (0.38) | 0.14 (0.35) | 0.18 (0.39) | 0.11 (0.32) |
| Congestive heart failure | 0.21 (0.41) | 0.17 (0.38) | 0.10 (0.30) | 0.10 (0.30) |
| Renal disease | 0.14 (0.35) | 0.11 (0.32) | 0.03 (0.16) | 0.05 (0.21) |
| Cancer | 0.12 (0.33) | 0.15 (0.36) | 0.25 (0.43) | 0.17 (0.37) |
| Liver disease | 0.00 (0.00) | 0.01 (0.11) | 0.01 (0.09) | 0.01 (0.08) |
| Psychiatric disease | 0.12 (0.33) | 0.06 (0.24) | 0.05 (0.22) | 0.06 (0.23) |
| COPD | 0.11 (0.31) | 0.15 (0.35) | 0.15 (0.35) | 0.14 (0.35) |
| Medication (mean [SD]) |  |  |  |  |
| Lipid lowering | 0.85 (0.36) | 0.89 (0.31) | 0.84 (0.37) | 0.82 (0.38) |
| Antihypertensive | 0.92 (0.27) | 0.95 (0.21) | 0.84 (0.37) | 0.82 (0.38) |
| Aspirin | 0.74 (0.44) | 0.78 (0.41) | 0.81 (0.39) | 0.79 (0.40) |
| Oral anticoagulant | 0.39 (0.49) | 0.34 (0.47) | 0.41 (0.49) | 0.27 (0.45) |
| ACE inhibitors | 0.39 (0.49) | 0.46 (0.50) | 0.27 (0.45) | 0.33 (0.47) |
| ARB | 0.44 (0.50) | 0.41 (0.49) | 0.35 (0.48) | 0.31 (0.46) |
| Alpha blockers | 0.05 (0.21) | 0.05 (0.21) | 0.03 (0.16) | 0.01 (0.11) |
| Beta blockers | 0.62 (0.49) | 0.59 (0.49) | 0.52 (0.50) | 0.46 (0.50) |
| Calcium channel blockers | 0.44 (0.50) | 0.55 (0.50) | 0.38 (0.49) | 0.40 (0.49) |
| Diuretics | 0.35 (0.48) | 0.42 (0.49) | 0.31 (0.46) | 0.28 (0.45) |
| Digoxin | 0.08 (0.27) | 0.03 (0.18) | 0.03 (0.16) | 0.02 (0.13) |

Supplementary table S6. Baseline characteristics of patients with chronic limb threatening ischemia (CLTI) undergoing endovascular treatment with or without drug eluting technologies, subdivided in groups with and without diabetes mellitus. DE=drug eluting, ACE = angiotensin converting enzyme, AMI = acute myocardial infarction, ARB = Angiotensin receptor blockers, COPD = chronic obstructive pulmonary disease, SD = standard deviation, USD = United States Dollars.

|  | Patients with diabetes mellitus | | Patients without diabetes mellitus | |
| --- | --- | --- | --- | --- |
|  | **DE methods** | **No DE methods** | **DE methods** | **No DE methods** |
| N | 201 | 1476 | 168 | 1508 |
| Age (mean [SD]) | 73.88 (9.78) | 75.22 (10.16) | 79.55 (9.55) | 79.26 (9.48) |
| Male sex (%) | 126 (62.7) | 892 (60.4) | 76 (45.2) | 605 (40.1) |
| Smoking status (%) |  |  |  |  |
| Non-smoker | 165 (82.1) | 1193 (80.8) | 104 (61.9) | 822 (54.5) |
| Smoker | 31 (15.4) | 249 (16.9) | 24 (14.3) | 331 (21.9) |
| Unknown | 5 (2.5) | 34 (2.3) | 40 (23.8) | 355 (23.5) |
| Civil status (%) |  |  |  |  |
| Married | 94 (46.8) | 648 (43.9) | 81 (48.2) | 520 (34.5) |
| Separated | 34 (16.9) | 313 (21.2) | 30 (17.9) | 311 (20.6) |
| Single | 26 (12.9) | 180 (12.2) | 13 ( 7.7) | 151 (10.0) |
| Widowed | 47 (23.4) | 334 (22.6) | 44 (26.2) | 523 (34.7) |
| Place of birth (%) |  |  |  |  |
| Sweden | 174 (86.6) | 1237 (83.8) | 155 (92.3) | 1328 (88.1) |
| Europe except Sweden | 11 (5.5) | 103 (7.0) | 4 (2.4) | 103 (6.8) |
| Outside Europe | 16 (8.0) | 136 (9.2) | 9 (5.4) | 77 (5.1) |
| Income per month after tax (USD) (mean [SD]) | 1968.48 (2554.47) | 1739.25  (1430.94) | 1857.90  (1693.29) | 1698.77  (1474.53) |
| Concomitant diseases (mean [SD]) |  |  |  |  |
| AMI | 0.26 (0.44) | 0.26 (0.44) | 0.21 (0.41) | 0.17 (0.37) |
| Coronary heart disease | 0.57 (0.50) | 0.50 (0.50) | 0.36 (0.48) | 0.35 (0.48) |
| Stroke | 0.21 (0.41) | 0.20 (0.40) | 0.11 (0.32) | 0.16 (0.36) |
| Any cardiovascular disease | 0.42 (0.49) | 0.41 (0.49) | 0.30 (0.46) | 0.29 (0.46) |
| Atrial fibrillation | 0.31 (0.47) | 0.34 (0.47) | 0.35 (0.48) | 0.29 (0.46) |
| Congestive heart failure | 0.38 (0.49) | 0.37 (0.48) | 0.31 (0.46) | 0.28 (0.45) |
| Renal disease | 0.25 (0.43) | 0.28 (0.45) | 0.10 (0.30) | 0.13 (0.34) |
| Cancer | 0.15 (0.36) | 0.18 (0.38) | 0.17 (0.38) | 0.19 (0.39) |
| Liver disease | 0.00 (0.00) | 0.02 (0.13) | 0.01 (0.08) | 0.01 (0.11) |
| Psychiatric disease | 0.12 (0.33) | 0.08 (0.28) | 0.06 (0.24) | 0.06 (0.24) |
| COPD | 0.12 (0.33) | 0.12 (0.33) | 0.15 (0.36) | 0.19 (0.39) |
| Medication (mean [SD]) |  |  |  |  |
| Lipid lowering | 0.79 (0.41) | 0.69 (0.46) | 0.60 (0.49) | 0.56 (0.50) |
| Antihypertensive | 0.94 (0.25) | 0.95 (0.23) | 0.88 (0.33) | 0.86 (0.34) |
| Aspirin | 0.68 (0.47) | 0.63 (0.48) | 0.66 (0.47) | 0.60 (0.49) |
| Oral anticoagulant | 0.47 (0.50) | 0.45 (0.50) | 0.42 (0.49) | 0.40 (0.49) |
| ACE inhibitors | 0.47 (0.50) | 0.46 (0.50) | 0.34 (0.47) | 0.35 (0.48) |
| ARB | 0.35 (0.48) | 0.36 (0.48) | 0.33 (0.47) | 0.25 (0.43) |
| Alpha blockers | 0.03 (0.17) | 0.04 (0.20) | 0.01 (0.08) | 0.02 (0.13) |
| Beta blockers | 0.62 (0.49) | 0.67 (0.47) | 0.54 (0.50) | 0.54 (0.50) |
| Calcium channel blockers | 0.40 (0.49) | 0.42 (0.49) | 0.30 (0.46) | 0.34 (0.47) |
| Diuretics | 0.64 (0.48) | 0.66 (0.47) | 0.59 (0.49) | 0.57 (0.49) |
| Digoxin | 0.03 (0.17) | 0.08 (0.27) | 0.08 (0.28) | 0.07 (0.25) |

Supplementary table S7. Hazard ratio (HR) with 95% confidence intervals (CI) for secondary outcomes in unadjusted and adjusted analysis for patients treated with drug eluting methods compared to those treated without drug eluting methods with intermittent claudication (IC) and chronic limb threatening ischemia (CLTI) with and without diabetes. MACE = major adverse cardiovascular events.

| Outcome | Model | Subgroup | HR (95% CI) | p value |
| --- | --- | --- | --- | --- |
| MACE | Adjusted | IC with diabetes | 1.296 (0.811-1.988) | 0.296 |
| MACE | Unadjusted | IC with diabetes | 0.965 (0.620-1.501) | 0.873 |
| MACE | Adjusted | IC without diabetes | 1.152 (0.783-1.694) | 0.472 |
| MACE | Unadjusted | IC without diabetes | 1.250 (0.853-1.832) | 0.251 |
| Total mortality | Adjusted | CLTI with diabetes | 0.763 (0.587-0.990) | 0.042 |
| Total mortality | Unadjusted | CLTI with diabetes | 0.745 (0.574-0.966) | 0.026 |
| Total mortality | Adjusted | CLTI without diabetes | 0.828 (0.613-1.119) | 0.219 |
| Total mortality | Unadjusted | CLTI without diabetes | 0.829 (0.615-1.118) | 0.218 |
| Amputation | Adjusted | CLTI with diabetes | 0.780 (0.568-1.072) | 0.125 |
| Amputation | Unadjusted | CLTI with diabetes | 0.766 (0.558-1.050) | 0.098 |
| Amputation | Adjusted | CLTI without diabetes | 1.033 (0.691-1.544) | 0.873 |
| Amputation | Unadjusted | CLTI without diabetes | 0.958 (0.643-1.429) | 0.834 |
